# Multi‐Modal Low‐Power Adaptive Braille Recognition System Based on Hf_0.3_Zr_0.7_O_2_ Memristor

**DOI:** 10.1002/advs.77601

**Published:** 2026-09-08

**Authors:** Jikang Xu, Jiacheng Wang, Ziye Li, Weidong Sun, Jianlin Wang, Qiang Lu, Wenjing Liu, Xiaoyang Liu, Yan Sun, Xiaobing Yan

**Affiliations:** ^1^ Key Laboratory of Brain‐Like Neuromorphic Devices and Systems of Hebei Province College of Electron and Information Engineering Hebei University Baoding People's Republic of China

**Keywords:** memristor, multimodal adaptive perception, reservoir computing

## Abstract

Tactile‐assisted perception systems hold great promise for robotics and human–machine interaction, yet current assistive systems for visually impaired individuals suffer from redundant data transmission, complex fusion algorithms, and high‐power consumption. These limitations can introduce millisecond‑scale processing latencies and restrict hardware‑level dynamic adaptability, posing substantial challenges for complex scenarios such as temperature‑induced tactile blurring. Here, we propose a multimodal adaptive perception system based on a Hf_0.3_Zr_0.7_O_2_ (HZO) epitaxial thin‐film ferroelectric memristor. The device features excellent ferroelectric properties with a remanent polarization of 2P_r_ ≈ 36.4 µC/cm^2^, stable 16‐state multilevel resistance, and robust synaptic plasticity for biomimetic emulation of biological learning rules. We developed adaptive sensing units integrating light, temperature, and pressure, which autonomously regulate response in dynamic environments. By encoding Braille digits into pulse sequences and adopting reservoir computing, we achieved a 91.65% maximum recognition accuracy for Braille digits 0–9. The system completes a single analog computation in ∼48 µs with only 8.51 nJ energy consumption, three orders of magnitude lower than conventional electronics. This work provides an efficient hardware strategy for neuromorphic perception and visually impaired assistive devices in complex environments.

## Graphical abstract

A multimodal adaptive perception system based on HZO ferroelectric memristor realizes efficient Braille digit recognition via reservoir computing. It delivers high recognition accuracy with ultralow‑energy analog computation, offering a promising hardware solution for environment‑robust neuromorphic assistive devices for visually impaired users.

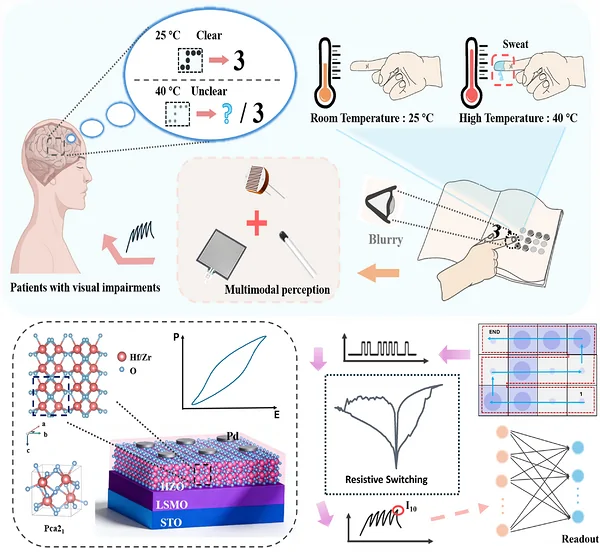

## 1 Introduction

**FIGURE 1:**
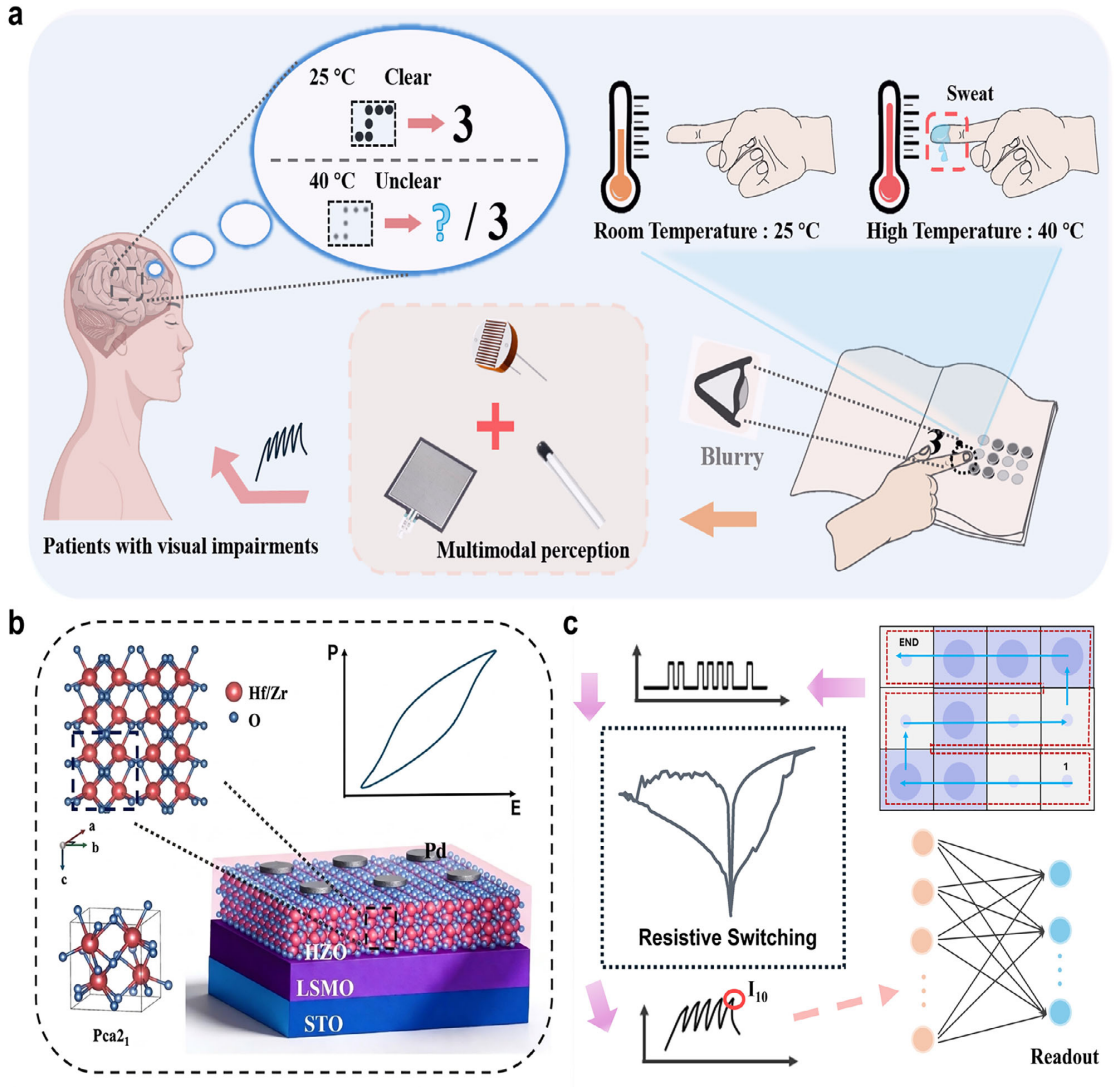
Multimodal adaptive perception system based on HZO ferroelectric memristors. (a) Simulation of behavior in visually impaired environments. (b) Integrated HZO ferroelectric memristors. (c) Recognition and classification of Braille numbers combined with reservoir computing.

Visually impaired individuals primarily acquire Braille information through fingertip‐based tactile perception [1]. Under ambient conditions, the fingers can accurately distinguish the spatial distribution of Braille dots, enabling the brain to reconstruct corresponding textual information. However, as environmental temperature increases, thermoreceptors in the human body trigger adaptive physiological responses, such as fingertip sweating, which reduces interfacial friction and weakens the contact force applied to Braille dots. This process ultimately leads to blurred tactile signals and degraded recognition accuracy, as illustrated in Figure 1a. This phenomenon highlights a critical challenge for tactile perception systems: maintaining operational stability and environmental adaptability under dynamically changing conditions [2, 3, 4].

Conventional assistive perception systems are typically built upon standard sensing modules and digital processing architectures, where information processing and decision‐making heavily rely on redundant data transmission and complex algorithmic fusion [5, 6, 7]. As a result, these systems often suffer from relatively high response latency (typically on the millisecond scale) and lack hardware‐level dynamic adaptability, making them unsuitable for complex scenarios in which temperature fluctuations distort tactile perception [8, 9, 10]. Consequently, the development of neuromorphic perception hardware characterized by environmental adaptability, low latency, and low power consumption has become an essential requirement for next‐generation assistive technologies serving visually impaired individuals [11, 12, 13, 14].

Over the past decade, memristors have emerged as promising building blocks for high‐performance neuromorphic systems due to their unique non‐volatile resistive switching characteristics and intrinsic in‐memory computing capability, which effectively overcome the limitations of conventional von Neumann architectures [10, 15, 16, 17, 18]. Among them, hafnium oxide‐based ferroelectric memristors have attracted particular attention because they combine excellent ferroelectric stability with low operating power while enabling precise emulation of synaptic weight plasticity and dynamic learning behaviors at the nanoscale [19, 20, 21, 22, 23, 24]. In contrast to conventional perovskite ferroelectric materials, which often suffer from severe polarization degradation—or even complete polarization loss—when scaled to ultrathin dimensions, hafnium oxide‐based ferroelectrics can maintain robust ferroelectricity even at thicknesses below 10 nm [19, 25, 26, 27]. This exceptional scalability provides a critical pathway toward device miniaturization and ultra‐high‐density integration, establishing hafnium‐based ferroelectrics as an attractive hardware platform for next‐generation adaptive neuromorphic computing systems [18, 28, 29, 30, 31, 32, 33].

In this work, we employ a Hf_0.3_Zr_0.7_O_2_ (HZO) epitaxial ferroelectric memristor that exhibits outstanding ferroelectric performance, including a remanent polarization of 2P_r_ ≈ 36.4 µC/cm^2^, 16 stable multilevel resistance states, and rich synaptic plasticity capable of emulating multiple biological learning rules, thereby providing a robust hardware foundation for localized adaptive processing of multimodal signals. Based on this high‐performance device, we further design and experimentally demonstrate a multimodal adaptive perception system that integrates optical, thermal, and pressure sensing functionalities, enabling dynamic response modulation and hardware‐level signal fusion in complex environments. By encoding Braille digits (0–9) into pulse sequences and combining the system with reservoir computing for pattern recognition, we achieve a recognition accuracy of 91.65%, with an ultralow analog computing latency of only ∼48 µs per operation and an average energy consumption of merely 8.51 nJ. The architecture of the fabricated HZO ferroelectric memristor is shown in Figure 1b, while Figure 1c illustrates the Braille digit classification process enabled by the device's non‐volatile resistive switching behavior in conjunction with reservoir computing. This work provides an efficient near‐sensor analog computing hardware strategy for neuromorphic perception systems and next‐generation assistive devices for visually impaired individuals operating in complex environments.

## 2 Results and Discussion

**FIGURE 2:**
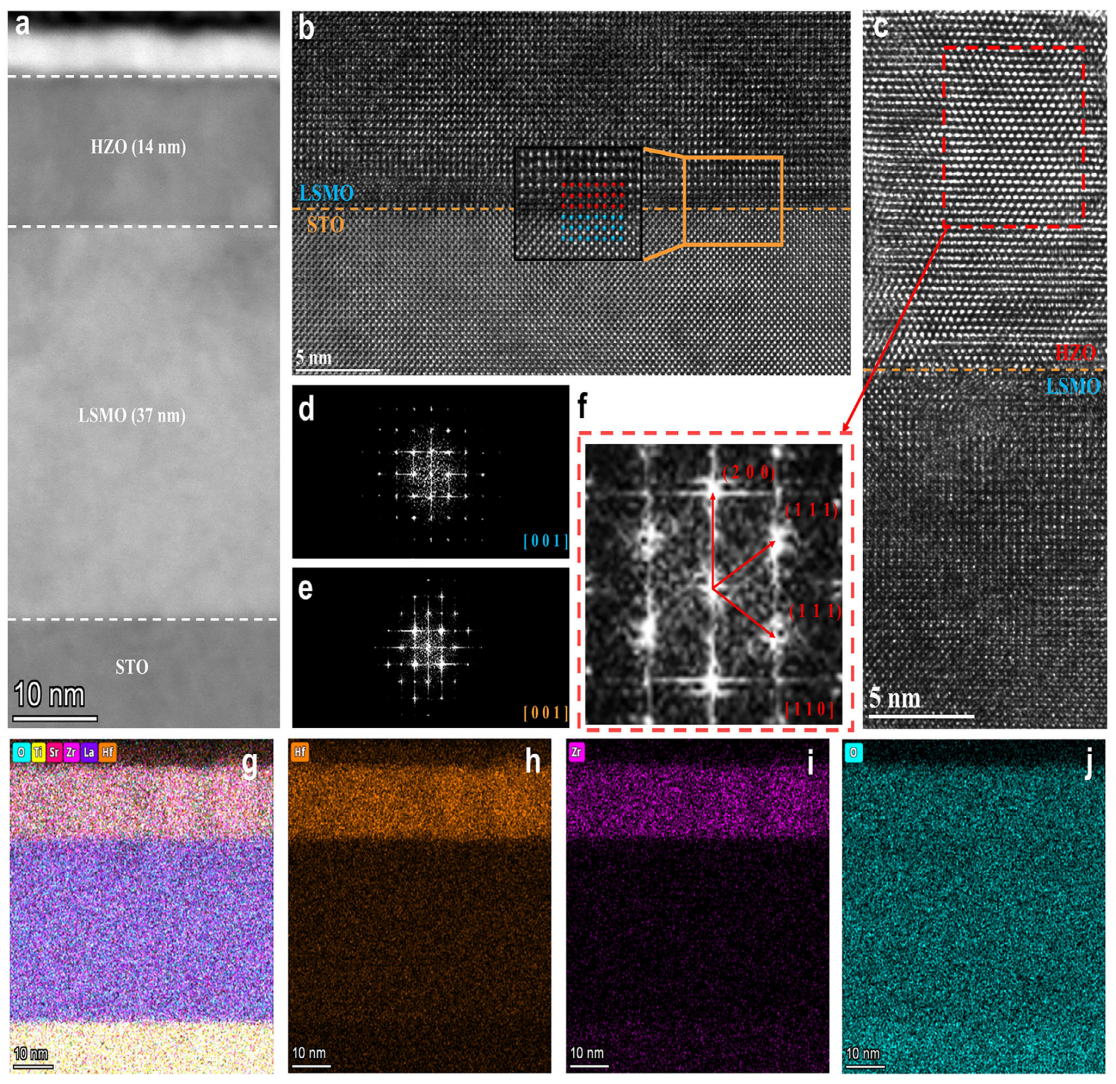
Microscopic characterization of HZO/LSMO/STO device structure. (a) TEM image of the cross‐section of the HZO/LSMO/STO device prepared at 850°C under HAADF. (b) TEM image of the STO substrate and LSMO lower electrode interface (the calibrated red and blue atoms show clear and consistent atomic arrangement). (c) TEM image of the HZO functional layer and LSMO lower electrode interface. (d–f) are the diffraction patterns after FFT calculation of selected areas of the STO substrate, LSMO lower electrode, and HZO functional layer, respectively. (g) The cross‐section corresponds to the EDS test. (h–j) are EDS elemental maps of Hf, Zr, and O elements, respectively.

First, the microstructure of the device was systematically characterized. A schematic illustration of the multilayer architecture of the HZO ferroelectric thin‐film memristor is presented in Figure 1b, consisting of a (001)‐oriented SrTiO_3_ (STO) substrate, an epitaxially grown La_0.67_Sr_0.33_MnO_3_ (LSMO) bottom electrode, a HZO ferroelectric functional layer, and a deposited Pd top electrode. The ferroelectricity of HZO is primarily associated with the orthorhombic phase belonging to the Pca2_1_ space group, in which the specific atomic arrangement breaks centrosymmetry, resulting in a separation between the centers of positive and negative charges and thereby generating spontaneous polarization. The inset of Figure 1b illustrates the atomic configuration of the ferroelectric orthorhombic HZO phase along the c‐axis, where three‐coordinated polar oxygen atoms are spatially separated by four‐coordinated nonpolar oxygen atoms within the O‐phase lattice. The crystallographic properties of HZO ferroelectric memristors fabricated at different deposition temperatures were further examined by X‐ray diffraction (XRD), as shown in Figure S1. Distinct diffraction peaks corresponding to well‐crystallized LSMO (001) and (002) planes can be clearly observed, indicating the formation of a high‐quality epitaxial heterostructure between LSMO and STO. In addition, a highly preferred diffraction peak appears at 2θ ≈ 30.2°, which corresponds to the (111) plane of HZO. These results suggest that the HZO functional layer deposited at 850°C forms a highly oriented ferroelectric phase while maintaining favorable lattice matching with the LSMO bottom electrode. To further elucidate the relationship between microstructure and ferroelectric performance, cross‐sectional transmission electron microscopy (TEM) characterization was conducted, with high‐resolution images presented in Figure 2. The high‐angle annular dark‐field (HAADF) TEM image in Figure 2a reveals the overall device cross‐section, where the thicknesses of the HZO and LSMO layers are approximately 14 and 37 nm, respectively.

The interfacial structure between STO and LSMO is shown in Figure 2b, where the clear and uniformly aligned atomic arrangement demonstrates the excellent crystallinity of the LSMO layer and confirms the high‐quality epitaxial relationship between the two layers. The corresponding fast Fourier transform (FFT) diffraction patterns of STO and LSMO are presented in Figure 2d,e, respectively. Since the interfacial quality between the HZO ferroelectric layer and the LSMO bottom electrode plays a critical role in determining device performance, this region was further examined using high‐resolution TEM, as shown in Figure 2c. The region highlighted by the red box in Figure 2c was selected for FFT analysis to further identify the crystallographic orientation of the HZO layer. As shown in Figure 2f, the obtained diffraction spots correspond to the orthorhombic phase of ferroelectric HZO, which is consistent with previously reported studies [17, 18]. Furthermore, energy‐dispersive X‐ray spectroscopy (EDS) elemental mapping of the device cross‐section is presented in Figures 2g–j and S2, clearly revealing the spatial distributions of Hf, Zr, O, La, Sr, Mn, and Ti. All elements exhibit uniform distributions with well‐defined layered interfaces, and no obvious elemental diffusion or aggregation is observed. This suppressed interfacial diffusion effectively minimizes its potential impact on electrical performance and provides a robust microstructural foundation for the stable operation of the device.

**FIGURE 3:**
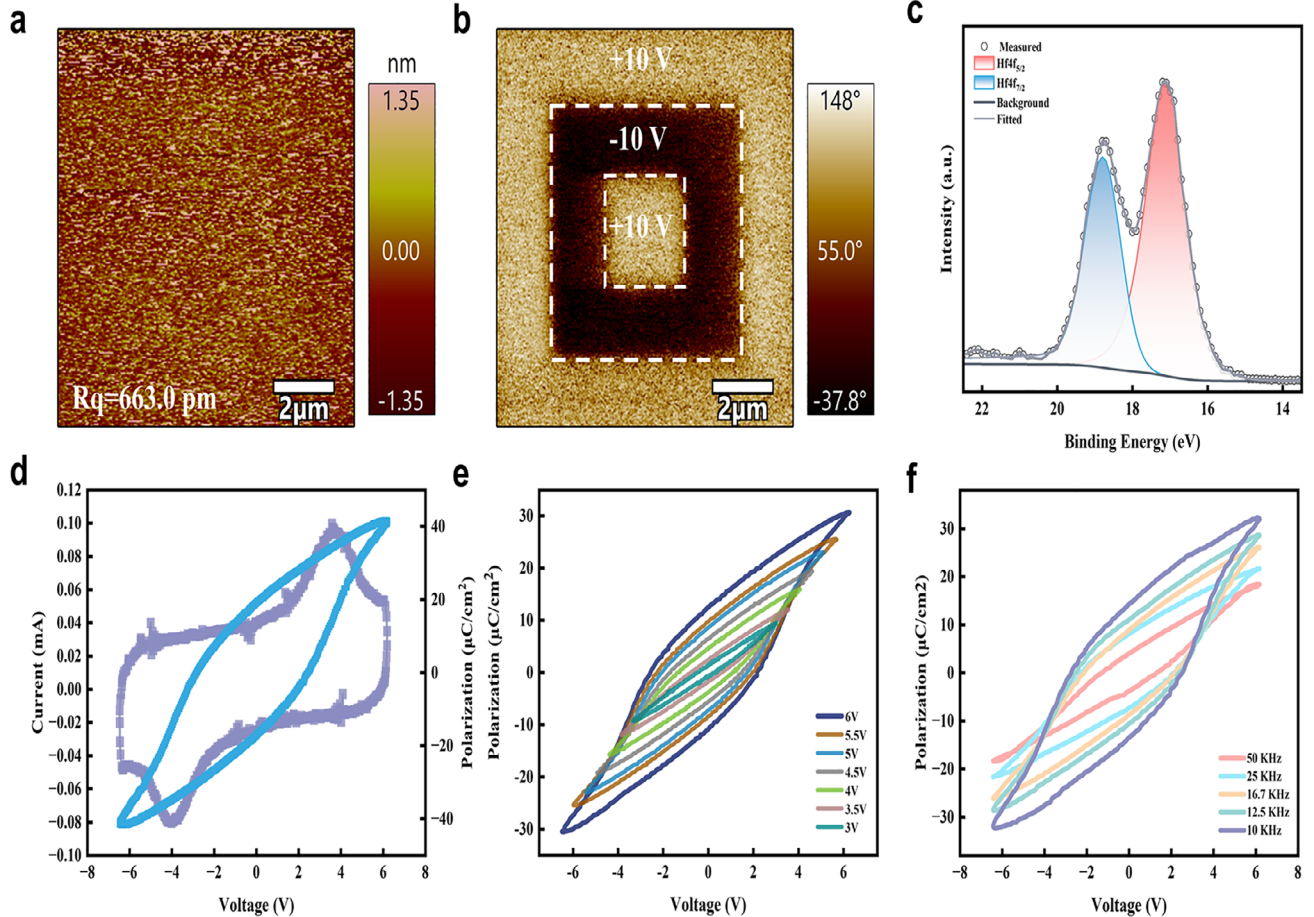
Physical characterization of Pd/HZO/LSMO/STO memristor. (a) AFM testing of HZO thin film. (b) Ferroelectric domain switching behavior of the HZO ferroelectric memristor under a bias voltage of 10 V. (c) XPS core spectra of Hf elements. (d) *P*–*V* loop (2Pr ∼ 36.4 µC/cm^2^) and displacement current of the HZO ferroelectric memristor. (e) *P*–*V* loop of the device with pulse voltage dependence. (f) *P*–*V* loop of the device with pulse frequency dependence.

The surface morphology of the HZO ferroelectric thin film was first characterized by atomic force microscopy (AFM). As shown in Figure 3a, the AFM image collected over a 10 µm × 10 µm area reveals a smooth and uniform film surface, with an ultralow root‐mean‐square roughness (Rq) of only 663.0 pm. Such a highly uniform surface provides a favorable interfacial condition for efficient ferroelectric domain switching. Ferroelectric domain behavior was further studied via piezoelectric‑response force microscopy (PFM). Upward and downward polarization states were induced by applying an opposite‑polarity external bias of ± 10 V over a 10 µm × 10 µm scanned region. Different bright‑dark domain patterns (Figure 3b), corresponding to distinct polarization directions and switching magnitudes, were observed, which directly confirms the reversible ferroelectric switching of HZO thin films. The chemical composition of the HZO film was further analyzed by x‐ray photoelectron spectroscopy (XPS). The core‐level spectra of the constituent elements are presented in Figures 3c and S3, confirming the expected elemental composition of the fabricated film. In addition, the surface morphology and elemental distributions of the HZO thin film were further characterized by scanning electron microscopy (SEM) and elemental mapping analysis, as shown in Figure S4. To further evaluate the ferroelectric properties of the device, polarization–voltage (*P*–*V*) measurements were conducted. As shown in Figures 3d and S5, the device exhibits stable bipolar polarization switching behavior, demonstrating robust ferroelectricity. Furthermore, pulse‐voltage‐dependent and frequency‐dependent ferroelectric measurements were performed, as presented in Figure 3e,f, respectively. The results indicate that the device possesses stable and tunable pulse‐voltage‐dependent characteristics, along with controllable multilevel polarization states.

**FIGURE 4:**
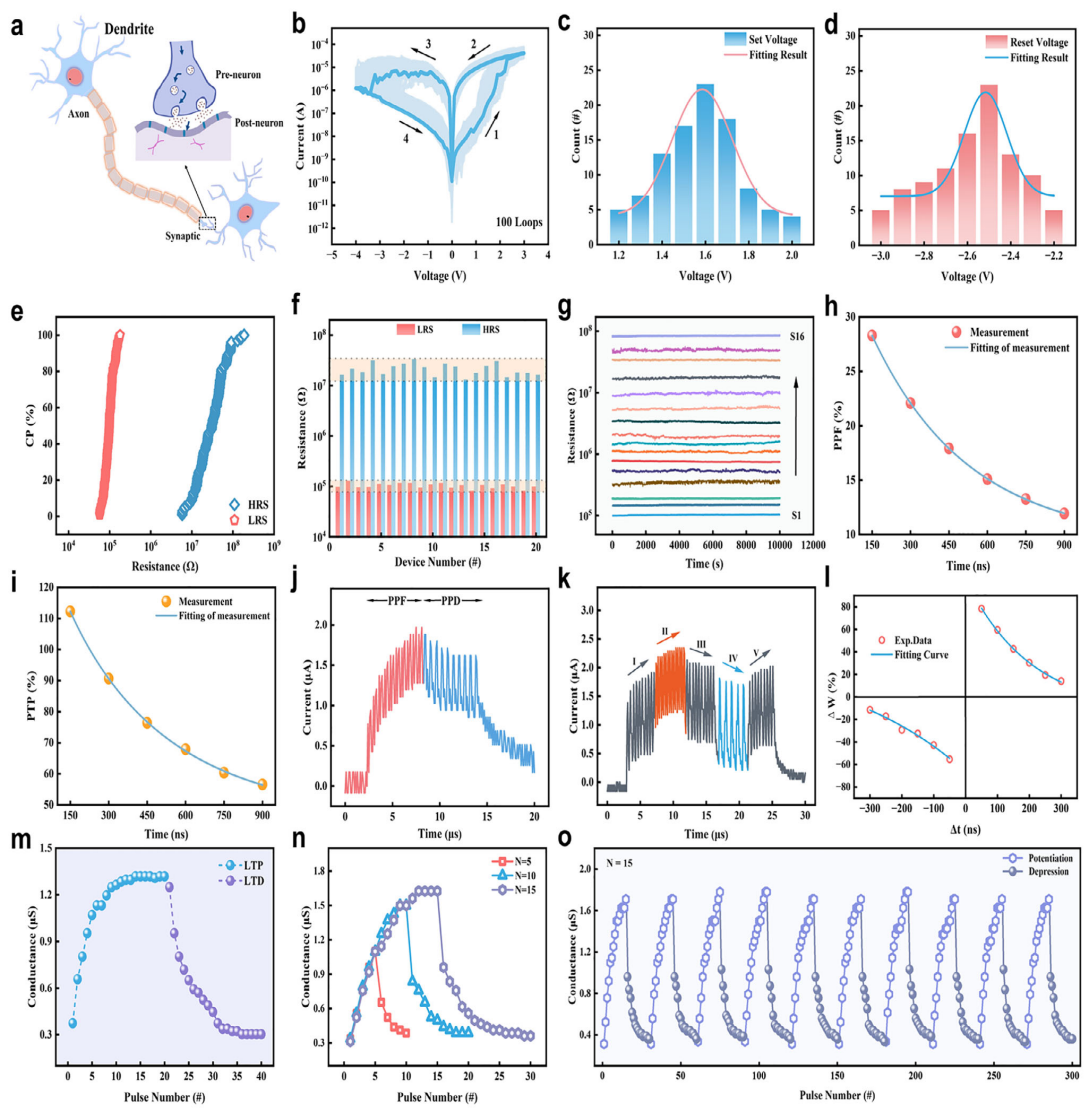
Electrical Characterization of HZO/LSMO/STO Devices. (a) Schematic diagram of neuron and synaptic structures. (b) 100 *I*–*V* test cycles of the device (the arrow indicates the voltage scanning direction). (c, d) Statistics of set and reset voltages of HZO memristors with Gaussian fitting. (e) Cumulative probability statistics of high and low resistance states in the device. (f) Distribution of high and low resistance states in 20 randomly selected devices. (g) Retention characteristics test of 16 resistance states in the device. (h) Paired‐pulse facilitation (PPF) index and fitting. (i) Post‐tetanic potentiation (PTP) index and fitting. (j) Simulation of the transition from PPF to PPD. (k) Spike rate‐dependent plasticity (SRDP) exhibited under different frequency stimuli. (l) Simulation of spike time‐dependent plasticity (STDP) learning rules based on HZO memristors. (m) Test results of long‐term potentiation/depression (LTP/LTD) behavior in the device. (n) Characteristics of synaptic enhancement and inhibition under different pulse numbers (*n* = 5, 10, 15). (o) Synaptic enhancement and inhibition over 10 cycles.

The brain functions as the central hub of the nervous system, where external stimuli are encoded into electrical signals by neurons and subsequently processed through synaptic transmission (Figure 4a). The dynamic modulation of synaptic weights, commonly referred to as synaptic plasticity, serves as the fundamental basis for neural computation and learning. Owing to their tunable conductance states, memristors can effectively emulate biological synaptic weights, providing a promising hardware platform for adaptive brain‐inspired computing. To systematically investigate the synaptic potential of the HZO memristor, comprehensive electrical characterizations were first conducted. Under direct current voltage sweeps, the device exhibited stable bipolar resistive switching behavior (Figures 4b and S6), with a high‐resistance state (HRS) of approximately 10^7^ Ω and a low‐resistance state (LRS) of approximately 10^5^ Ω, corresponding to an ON/OFF ratio exceeding two orders of magnitude. The set and reset voltages showed narrow statistical distributions that could be well fitted by Gaussian functions (Figure 4c,d). During cycling measurements, the device demonstrated high yield and extremely low operational variation (Figure 4e,f). In addition, the device exhibited excellent multilevel retention characteristics, where 16 distinct conductance states remained stable for over 10^4^ s (Figure 4g) without obvious degradation after 10^5^ switching cycles (Figure S6c), and *I*–*V* test results of different devices showed no significant change (Figure S7). To further clarify the resistive switching mechanism, the current–voltage characteristics were fitted in Figure S8, revealing three distinct transport regimes corresponding to space‐charge‐limited current (SCLC), thermionic emission (TE), and trap‐assisted tunneling (TAT), respectively. These results suggest that the resistive switching behavior likely originates from the synergistic effect of ferroelectric polarization and oxygen vacancies [34]. Notably, the presence of defect‐related oxygen signals in Figure S3b further indirectly confirms the existence of oxygen vacancies in the HZO film.

The neuromorphic functionalities of the device were further evaluated through pulse modulation experiments. Continuous conductance tuning was achieved by varying pulse amplitude, pulse width, and pulse interval (Figure S9), successfully emulating excitatory postsynaptic current (EPSC) behavior and its relaxation dynamics (Figure S10). The device also exhibited paired‐pulse facilitation (PPF) and post‐tetanic potentiation (PTP), where the facilitation index as a function of pulse interval could be well fitted using a double‐exponential function (Figure 4h,i). In frequency‐dependent measurements, the device demonstrated a transition from paired‐pulse facilitation to paired‐pulse depression (Figure 4j) and successfully emulated refractory behavior as well as the transition from short‐term memory (STM) to long‐term memory (LTM) (Figures S11 and S12). Furthermore, multiple synaptic learning rules were reproduced, including spike‐rate‐dependent plasticity (SRDP), spike‐duration‐dependent plasticity (SDDP), and spike‐amplitude‐dependent plasticity (SADP) (Figures 4k and S13). Collectively, these results demonstrate that the HZO memristor can effectively emulate the dynamic plasticity of biological synapses, providing a robust hardware foundation for adaptive neuromorphic systems.

Spike‐timing‐dependent plasticity (STDP), a key learning mechanism based on Hebbian theory, was further investigated by modulating the temporal interval between pre‐ and postsynaptic spikes. As shown in Figure 4l, when the time interval is positive (i.e., the presynaptic spike precedes the postsynaptic spike), synaptic potentiation occurs. In contrast, when the time interval becomes negative, synaptic depression is observed. The fitting results confirm that the device can reliably emulate the STDP learning rule. Benefiting from the reversible conductance modulation of the HZO ferroelectric memristor, long‐term potentiation/depression (LTP/LTD) behaviors were further demonstrated. Under pulse sequences of ± 4 V and 150 ns, the device conductance gradually increased from 0.37 to 1.38 µS during the LTP process and subsequently decreased to 0.30 µS during the LTD process (Figure 4m). By further varying the pulse number (5, 10, and 15 pulses) and pulse intervals, the device exhibited stable, reproducible, and tunable conductance modulation behaviors (Figures 4n,o and S14). Moreover, a pulse‐interval‐based programming strategy was employed to successfully emulate basic arithmetic operations (Figure S15). These results collectively demonstrate that the HZO synaptic device possesses stable and precise bidirectional conductance modulation capabilities, together with rich synaptic plasticity, making it a promising candidate for high‐performance neuromorphic circuits and adaptive perception systems.

**FIGURE 5:**
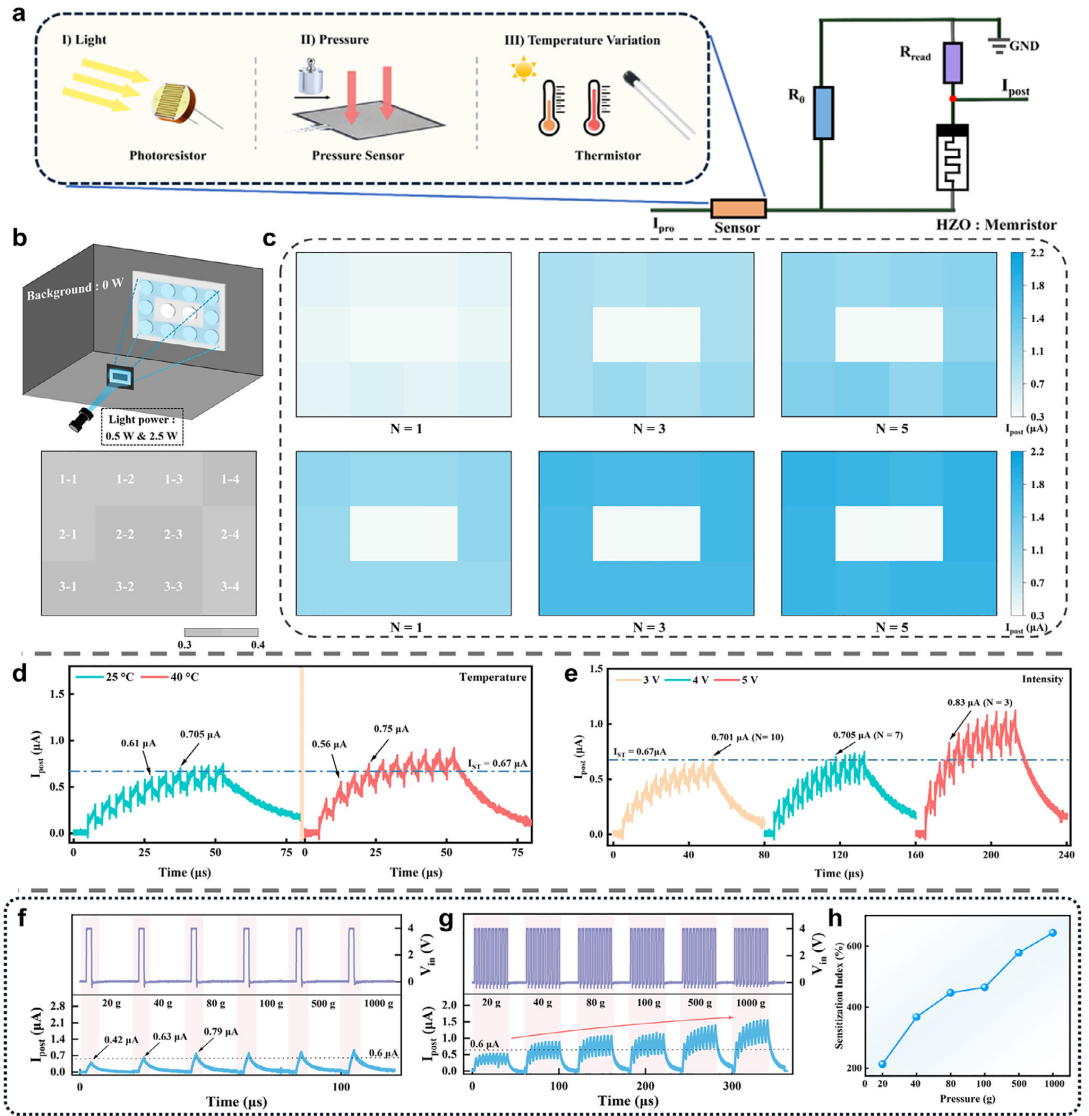
Adaptive sensing unit based on HZO ferroelectric memristor. (a) Circuit schematic of the adaptive sensing unit, specifically for light, temperature, and pressure. (b) Schematic diagram of dark adaptation test, with the lower part showing the initial current value of the device in a dark environment. (c) Dark adaptation behavior, achieving the perception of rectangular images. (d) Human sweat conditions in room temperature and high temperature environments, with the temperature sensing unit's response more prone to breaking the sweat threshold at high temperatures. (e) Human sweat conditions under different exercise intensities, with pulse amplitudes of 3, 4, and 5 V, respectively. (f–h) Simulation of nociceptor behavior.

Inspired by biological sensory adaptation mechanisms, we developed three adaptive perception modules for light, temperature, and pressure sensing by integrating HZO ferroelectric memristors with photosensitive, thermosensitive, and piezoresistive sensors, respectively. The circuit architecture of these adaptive sensing units is illustrated in Figure 5a. These modules were designed to emulate the in situ signal acquisition and dynamic response behaviors of biological sensory systems under external stimuli. For the light perception unit, dynamic responses to varying illumination intensities were achieved by optimizing device parameters and introducing a current‐limiting resistor (R_0_). As the light intensity increased, the terminal voltage gradually rose and eventually reached saturation, exhibiting a stimulus‐dependent desensitization behavior (Figure S16). Furthermore, a 3 × 4 memristor arrays operating under dark and bright background conditions was employed to mimic the light/dark adaptation process of the human visual system. As the number of adaptive pulses (N) increased, target patterns, such as square and rectangular shapes, gradually became distinguishable, demonstrating adaptive visual enhancement capabilities (Figures 5b,c and S17).

The temperature perception unit was designed to emulate adaptive regulation associated with human sweating behavior. A sweating current threshold of 0.67 µA was defined to characterize this process. When the ambient temperature increased, or the simulated “exercise intensity” (represented by pulse amplitude) was enhanced, the response current exceeded the threshold at an earlier stage (Figure 5d,e). In addition, pulse width and pulse interval were modulated to mimic the effects of exercise duration and environmental humidity on sweating behavior, respectively (Figures S18 and S19). The pressure perception unit was designed to emulate nociceptive responses to harmful mechanical stimuli by defining a pain current threshold (Ith = 0.6 µA) and its corresponding threshold voltage (Vth) (Figure S20a,b). It was observed that applying sub‐threshold pulses after an initial stimulation pulse induced a sensitization effect, manifested as an enhanced response current, whereas increasing the pulse interval led to desensitization behavior (Figure S20c–f). After integrating a thin‐film pressure sensor, the output current of the system exhibited a positive correlation with the applied pressure (Figure S21). When the applied pressure exceeded 40 g, the response current surpassed Ith, thereby mimicking biological pain perception. Increasing the number of stimulation pulses further amplified this nociceptive response (Figures 5f–h and S22). Collectively, these results demonstrate that the HZO memristor‐based adaptive sensing units can effectively emulate multimodal biological sensory adaptation behaviors, providing a critical hardware foundation for constructing environment‐adaptive neuromorphic perception systems.

**FIGURE 6:**
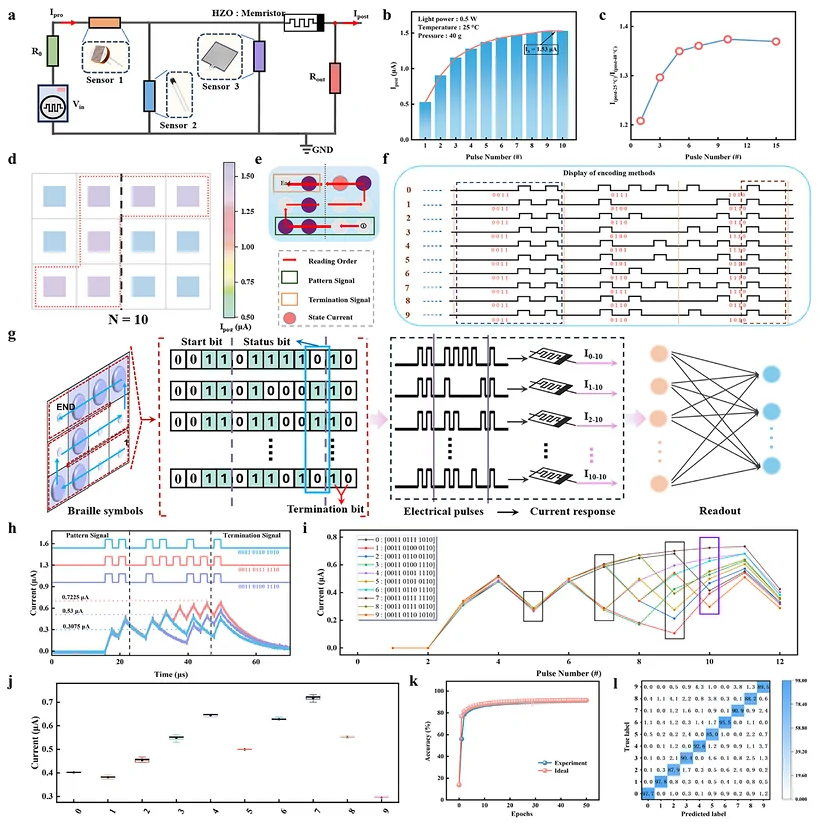
Multimodal Adaptive Perception System. (a) Circuit schematic of the multimodal adaptive perception system. (b) Pulse characteristic test. (c) Current response ratio of the system under room temperature and high temperature environments. (d) Pattern enhancement recognition training, *n* = 10, with an ambient temperature of 25°C. (e) Schematic diagram of the digital braille programming pulse scheme. (f) Adaptive pulse programming scheme corresponding to braille numbers 0–9. (g) Schematic diagram of data flow during the system testing process. (h) The reservoir current value of the example image. (i) The two feature point positions selected for the reservoir state in the memristor. (j) The distribution of reservoir states in the memristor. (k) The recognition accuracy during the training process. (l) The pseudo‐color confusion matrix shows the classification results obtained from the RC system through experiments on the expected output.

Based on the characterization of individual sensing channels, we further integrated optical, thermal, and pressure sensing modules to construct a multimodal adaptive perception system. As illustrated in Figure 6a, the HZO ferroelectric memristor‐based system is designed to emulate the adaptive perception behavior of visually impaired individuals under complex environmental conditions, with potential applications in assistive technologies. The detailed circuit configuration and physical connections are provided in Figure S23. In this architecture, the postsynaptic current (Ipost) generated by the memristor serves as the state variable for subsequent pattern recognition and algorithmic processing. The pulse response characteristics of the multimodal adaptive circuit were first evaluated. As shown in Figure 6b, the system exhibits stable pulse‐dependent electrical responses. To investigate its environmental adaptability, the current response ratios under two different temperature conditions were compared in Figure 6c. The current ratio gradually increased with pulse number and eventually reached a stable state, preliminarily demonstrating the capability of the system to mimic adaptive tactile perception in visually impaired users. The corresponding current responses under different temperatures are further presented in Figure S24.

Braille is a tactile writing system specifically designed for visually impaired individuals, where linguistic information is conveyed through the spatial arrangement of raised dots. As the smallest information unit in Braille, different combinations of raised and recessed dots represent distinct semantic meanings. Figure S25 presents the numerical symbols used in the current Chinese Braille system. Unlike conventional six‐dot Braille, Chinese Braille is optimized according to the characteristics of the Chinese language and adopts a 3 × 4 dot‐matrix architectures, consisting of twelve discrete tactile positions. The raised dots are highlighted by large red and purple markers in the schematic. Based on the spatial distribution characteristics of Braille digits, a memristor array was employed to emulate the tactile pattern recognition process of visually impaired users while also validating device‐to‐device consistency. Figures 6d and S26 illustrate the training evolution process for Braille digit “3” under room‐temperature conditions. As the number of adaptive pulses increased, the postsynaptic current (Ipost) gradually increased, resulting in clearer pattern reconstruction and improved distinguishability. Moreover, each training iteration produced noticeably enhanced recognition performance compared with the previous cycle. The corresponding enhancement process under elevated temperature conditions is shown in Figure S27. Inspired by the spatial distribution of Braille dot arrays shown in Figure S25, we further developed a Braille traversal and encoding strategy based on memristor postsynaptic current responses.

As illustrated in Figure 6e, the Braille reading sequence follows a predefined traversal order and can be interpreted as a combination of three four‐bit binary sequences. The first four bits of each pulse sequence were fixed as 0011, serving as a start code that triggers the classification process for Braille digits (0–9). The final two bits (10) were defined as a termination code to stop the classification process, while the intermediate six bits were used as adaptive state codes for signal extraction and analysis. Specifically, the abstract Braille dot distribution information is quantitatively converted into standardized electrical pulse sequences via artificial electrical encoding, and the dynamic current responses of the single memristor device excited by different pulse sequences are captured and defined as the final reservoir state, realizing the one‐to‐one mapping from input Braille information to physical reservoir states. The corresponding pulse encoding schemes for Braille digits 0–9 are shown in Figure 6f,g. The postsynaptic current generated during the 10th pulse for each digit was extracted as the reservoir state and subsequently fed into the classification framework for pattern recognition. After applying the programming pulse sequences corresponding to Braille digits 0–9, the current responses of the multimodal adaptive perception system are shown in Figures 6h and S28. Notably, a single analog computing cycle requires only 48 µs, corresponding to the total duration of the pulse sequence. The average energy consumption of the circuit was calculated using Equation (1):

(1) $$P=\frac{\sum_{1}^{10}UI_{n}t_{n}}{10}\;\left(n=0,1,2\text{…},9\right)$$

where U represents the input pulse voltage (6 V), I_n_ denotes the total system current after pulse stimulation, and t_n_ corresponds to the pulse duration at the high‐voltage state. Statistical analysis indicates that the average energy consumption of the proposed system is only 8.51 nJ, which is approximately three orders of magnitude lower than that of conventional electronic systems (Table S1). Reservoir‐state distributions are visualized in Figure 6i,j, exhibiting well‐separated state clusters for different Braille digits. Built upon Python‐based numerical simulation, the output layer utilizes the multi‐level resistive‐switching behavior of HZO memristors to map distinct device conductance states onto synaptic weights, and this configuration enables high‐precision feature classification targeting ten Braille digits (0–9), while the corresponding weight distribution of the constructed output layer is provided in Figure S29a. After training, the system achieved a maximum recognition accuracy of 91.65% (Figures 6k and S29b). Furthermore, the pseudocolor confusion matrix in Figure 6l demonstrates that the reservoir computing system can accurately distinguish all ten Braille digits, further validating the effectiveness of the proposed hardware‐based recognition framework.

## 3 Conclusions

In this work, we fabricated a Pd/HZO/LSMO/STO ferroelectric memristor that exhibits excellent ferroelectric performance, including a remanent polarization of 2P_r_ ≈ 36.4 µC/cm^2^, endurance exceeding 10^3^ switching cycles, and 16 stable multilevel resistance states with retention times longer than 10^4^ s. The device also demonstrated reliable emulation of diverse synaptic plasticity behaviors, including STDP, SRDP, PPF, and LTP/LTD, providing a robust hardware foundation for adaptive learning. Building upon this device platform, we further integrated optical, thermal, and pressure sensors to construct a multimodal adaptive perception system for Braille recognition. By combining pulse encoding with reservoir computing, the proposed system achieved high‐accuracy recognition of Braille digits from 0 to 9, reaching a classification accuracy of 91.65%. Our results demonstrate that the HZO ferroelectric memristor‐enabled near‐sensor analog computing architecture can efficiently perform multimodal information fusion and pattern recognition in complex environments with ultralow latency (∼48 µs per operation) and ultralow energy consumption (8.51 nJ per operation). This work provides an energy‐efficient and environment‐adaptive hardware strategy for next‐generation assistive technologies for visually impaired individuals, while also offering broader opportunities for neuromorphic perception systems, intelligent robotics, and adaptive human–machine interfaces.

## 4 Experimental Section

### 4.1 Fabrication of Pd/HZO/LSMO/STO/Si Devices

In this study, ferroelectric memristors based on Hf_0.3_Zr_0.7_O_2_ were prepared by pulsed laser deposition technology. La_0.67_Sr_0.33_MnO_3_ (LSMO) films were epitaxially grown on SrTiO_3_(100) substrates in an atmosphere of 750°C and 195.5 mtorr. HZO films were epitaxially grown on LSMO at three different temperatures (750°C, 800°C, and 850°C) using a Physik COMPex Pro 20 S KrF excimer laser (λ = 248 nm) at an oxygen atmosphere of 75 mtorr, a laser energy density of 1.3 J/cm^2^, a frequency of 2 Hz, and cooled to room temperature at a rate of 5°C/min in a hyperoxic atmosphere to prepare a 14nm HZO thin film. Finally, a 30nm Pd top electrode was prepared by magnetron sputtering in an argon flow rate of 20 SCCM and 1 Pa for 12 min.

### 4.2 Characterization and Measurement

The X‐ray diffraction (XRD) spectrum was measured by a diffractometer. Transmission electron microscopy (TEM) was performed using a Fei Tecnai G2 F20 ST FE‐TEM. The surface morphology was observed by atomic force microscopy (Cipher‐S, Asylum Research, GAI‐SA). The electrical characteristics and circuit testing of the memristor were conducted using a Keithley 2400 source meter, Keysight B1500A semiconductor parameter analyzer, RIGOL DG5072 waveform generator, and RIGOL MSO4034 digital oscilloscope.

## Author Contributions

X.Y. conceived the research idea, designed the overall experimental scheme, and supervised the whole research work. J.X. and J.W. performed experiments, collected raw data, analyzed the experimental results, completed device testing, drafted the original manuscript, and designed application scenarios for the proposed system. Z.L., Q.L., and W.L. participated in data testing and experimental verification. W.S. and J.W. conducted partial circuit tests. X.L. and Y.S. processed data and drew figures. All authors discussed the experimental findings, evaluated the research value, and approved the revised manuscript for final submission.

## Conflicts of Interest

The authors declare no conflicts of interest

## Supporting information




**Supporting File**: advs77601‐sup‐0001‐SuppMat.docx.

## Data Availability

The data that support the plots within this paper and other findings of this study are available from the corresponding authors upon reasonable request.

## References

[1] X. Zheng , R. Zhang , Y. Shi , et al., “Structure‐Dependent Resonant Frequency Engineering of Textile Tactile Sensors toward Rapid and Precise Braille Recognition Surpassing Human Sensation,” Advanced Science 13, no. 13 (2026): 20152. 10.1002/advs.202520152 PMC12955891 41527453

[2] S. Zhang , J. Du , Y. Sun , et al., “A Braille Detection System Based on Visuo‐Tactile Sensing,” Measurement 247 (2025): 116827.

[3] Z. Qin , G. Shen , J. Jiang , et al., “Near‐Sensor Reservoir Computing for Braille Recognition via High Stability Memristors,” Applied Physics Letters 126 (2025): 093504.

[4] C. Yang , H. Wang , Z. Cao , et al., “Memristor‐Based Bionic Tactile Devices: Opening the Door for Next‐Generation Artificial Intelligence,” Small 20 (2024): 2308918. 10.1002/smll.202308918 38149504

[5] B.‐S. Park , S.‐M. Im , H. Lee , et al., “Visual and Tactile Perception Techniques for Braille Recognition,” Micro and Nano Systems Letters 11, no. 1 (2023): 23.

[6] X.‐F. Zhao , C.‐Z. Hang , H.‐L. Lu , et al., “A Skin‐Like Sensor for Intelligent Braille Recognition,” Nano Energy 68 (2020): 104346.

[7] A. Antonacopoulos and D. Bridson , “A Robust Braille Recognition System,” in Document Analysis Systems VI: International workshop on document analysis systems (DAS 2024) (Springer, 2004), 533–545.

[8] J. Zhao , C. S. Jorgensen , K. Mahalingam , et al., “High‐Temperature Memristors Enabled by Interfacial Engineering,” Science 392, no. 6799 (2026): 771–779. 41886537 10.1126/science.aeb9934

[9] R. Zhao , T. Wang , T. Moon , et al., “A Spiking Artificial Neuron Based on one Diffusive Memristor, one Transistor and one Resistor,” Nature Electronics 8, no. 12 (2025): 1211–1221.

[10] Z. Wang , W. Song , T. Wang , et al., “Real‐Time Signal Processing Enabled by Fused Networks on a Memristor‐Based System on a Chip,” Science Advances 11, no. 30 (2025): adv3436. 10.1126/sciadv.adv3436 PMC12292901 40712027

[11] J. Xu , Y. Zhang , J. Wang , et al., “Ultra‐Low Power and Robust Bi_2_SeO_5_ Films Optoelectronic Memristors for Bio‐Visual Perception Computing Systems,” Advanced Materials 37, no. 40 (2025): 2509174. 10.1002/adma.202509174 40696763

[12] X. Zhang , M. Deng , Y. Luo , Z. Zhao , X. Tan , and X. Fang , “Self‐Powered Phototriggered Memristor Array with pW‐Level Computing for Monolithic in‐Sensor Vision,” Advanced Materials 38, no. 17 (2026): 23017. 10.1002/adma.202523017 41721621

[13] K. Gao , B. Sun , Z. Cao , et al., “Brain‐Inspired Computing Based on Large‐Scale Memristor Crossbar Arrays,” Advanced Functional Materials 36, no. 32 (2026): 28309.

[14] M. Wang , J. Tu , Z. Huang , et al., “Tactile Near‐Sensor Analogue Computing for Ultrafast Responsive Artificial Skin,” Advanced Materials 34, no. 34 (2022): 2201962. 10.1002/adma.202201962 35816720

[15] K. Liu , S. Tan , Z. Fan , et al., “In‐Sensor Image Memorization, Low‐Level Processing, and High‐Level Computing by Using Above‐Bandgap Photovoltages,” Nature Communications 17, no. 1 (2025): 408. 10.1038/s41467-025-67103-x PMC12796322 41387710

[16] H. Lin , J. Ou , Z. Fan , et al., “In Situ Training of an in‐Sensor Artificial Neural Network Based on Ferroelectric Photosensors,” Nature Communications 16, no. 1 (2025): 421. 10.1038/s41467-024-55508-z PMC11707328 39774072

[17] Y. Fan , S. Zhang , Z. Xue , et al., “Hidden Structural Phase Transition Assisted Ferroelectric Domain Orientation Engineering in Hf_0.5_Zr_0.5_O_2_ Films,” Nature Communications 16, no. 1 (2025): 4232. 10.1038/s41467-025-59519-2 PMC12059023 40335512

[18] X. Yan , X. Jia , Y. Zhang , et al., “A Low‐Power Si:HfO_2_ Ferroelectric Tunnel Memristor for Spiking Neural Network,” Nano Energy 107 (2023): 108091.

[19] H. Meng , X. Ren , S. Li , et al., “Epitaxial Yttrium Doped Hafnia with Giant Remnant Polarization for Ferroelectric Tunnel Junction Artificial Synapses and Neuromorphic Computing,” ACS Nano 20 (2026): 11032–11043. 41902782 10.1021/acsnano.5c20633

[20] Y. Wang , et al., “A Stable Rhombohedral Phase in Ferroelectric Hf(Zr)_1+x_O_2_ Capacitor With Ultralow Coercive Field,” Science 381, no. 6657 (2023): 558–563. 37535726 10.1126/science.adf6137

[21] B. Bakhit , X. Xie , S. M. Fairclough , et al., “HfO_2_‐Based Memristive Synapses with Asymmetrically Extended p‐N Heterointerfaces for Highly Energy‐Efficient Neuromorphic Hardware,” Science Advances 12, no. 12 (2026): aec2324. 10.1126/sciadv.aec2324 PMC13004029 41861000

[22] Y. Zhang , S. Yan , Y. Zhu , et al., “Flexible Zr‐Doped Hafnium Oxide Ferroelectric Memcapacitive Synaptic Devices for Neuromorphic Computing,” Advanced Composites and Hybrid Materials 8, no. 4 (2025): 278.

[23] W. Park , G. Kim , H. Chae , S. Lee , and S. Kim , “HfAlOx‐Based Optical Ferroelectric Memristor with Transparent Electrode for RGB Color Image Classification via Physical Reservoir,” Nano Energy 142 (2025): 111190.

[24] G. An , S. Lee , Y. Seo , and S. Kim , “Multifunctional Ferroelectric Synaptic Memristors Based on HfAlO X with Enhanced Pavlovian Learning and Physical Reservoir Computing Systems,” Physical Chemistry Chemical Physics 27, no. 45 (2025): 24522–24533. 41196072 10.1039/d5cp03132j

[25] K.‐F. Luo , Z. Ma , D. Sando , Q. Zhang , and N. Valanoor , “Hybrid Ferroelectric Tunnel Junctions: State of the Art, Challenges, and Opportunities,” ACS nano 19, no. 7 (2025): 6622–6647. 39937054 10.1021/acsnano.4c14446

[26] M. Lederer and T. Kämpfe , “Synaptic Devices Based on Ferroelectric Hafnium Oxide: Recent Advances, Challenges, and Future Perspectives,” Applied Physics Letters 126 (2025): 130503.

[27] L. Carpentieri , T. Mikolajick , and S. Slesazeck , “Effect of HZO Thickness Scaling in the Bilayer Ferroelectric Tunnel Junction,” ACS Applied Electronic Materials 7, no. 11 (2025): 5008–5017. 40520484 10.1021/acsaelm.5c00469 PMC12160526

[28] Z. Zhou , L. Li , Y. Feng , et al., “Advancing the Frontiers of HfO_2_‐Based Ferroelectric Memories: Innovative Concepts from Materials to Applications,” Advanced Materials 37, no. 50 (2025): 09525. 10.1002/adma.202509525 PMC12710576 41178162

[29] C.‐Y. Teng , C.‐W. Hsu , C.‐H. Chang , et al., “Oxygen Migration Impact on Ferroelectric Evolution in Hf_0.5_Zr_0.5_O_2_ Devices,” Applied Physics Letters 126, no. 19 (2025): 193502.

[30] Y. Lee and S. Lee , “Polarization‐Controlled Memristive Synapse Characteristics of HfZrO_2_‐Based Ferroelectric Switchable Diode,” Journal of Alloys and Compounds 1038 (2025): 182700.

[31] C. F. Chirila , G. A. Boni , D. G. Popescu , et al., “Ferroelectric Hf_0.5_Zr_0.5_O_2_ Thin Films on TiN/Si Substrates Grown by Pulsed Laser Deposition at CMOS‐Compatible Temperatures,” Ceramics International 51, no. 26 (2025): 50941–50950.

[32] H. Yang , M. Duan , C. Kang , G. Yang , W. Zhang , and C. Jia , “Imitation of a Dual‐Modal Synapse Based on a Hf_0.5_Zr_0.5_O_2_ Ferroelectric Tunnel Junction for Neuromorphic Computing,” ACS Applied Electronic Materials 6, no. 10 (2024): 7591–7599.

[33] T. Liang , M. Chi , Y. Zhao , et al., “High‐Performance Artificial Synapse Developed by HZO on (110) NSTO,” ACS Applied Nano Materials 7, no. 16 (2024): 19006–19013.

[34] Y. Kim , Y. J. Lee , J. Yang , et al., “Programmable Ferroelectric Rectifier for Reliable and Efficient Neuromorphic Crossbar Array,” Nature Communications 17 (2026): 6377. 10.1038/s41467-026-70727-2 PMC13376401 41844658

