# Supplementary material for: Multi‐Modal Low‐Power Adaptive Braille Recognition System Based on Hf0.3Zr0.7O2 Memristor
Source: Adv Sci (Weinh). 2026 Sep 8:e77601. Online ahead of print. doi: 10.1002/advs.77601 (PMC13554378; doi:10.1002/advs.77601)
Supplement: Supplementary file 1 [file ADVS-9999-e77601-s001.docx]

**Supplementary Information**

**Multi-Modal Low-Power Adaptive Braille Recognition System Based on Hf_0.3_Zr_0.7_O_2_ Memristor**

*Jikang Xu^1,#^, Jiacheng Wang^1,#^, Ziye Li ^1^, Weidong Sun ^1^, Jianlin Wang ^1^, Qiang Lu ^1^,* *Wenjing Liu ^1^, Xiaoyang Liu ^1^, Yan Sun ^1^, Xiaobing Yan^1,*^*

^1^Key Laboratory of Brain-Like Neuromorphic Devices and Systems of Hebei Province, College of Electron and Information Engineering, Hebei University, Baoding 071002, P. R. China.

* Correspondence should be addressed to Xiaobing Yan, xiaobing_yan@126.com

#These authors contributed equally to the work

**This file includes:**

**Supplementary Figs. S1 to S29**

**Table S1**

**Figure S1. The cross-section corresponds to the EDS test.** (**a**) EDS elemental maps of La. (**b**) EDS elemental maps of Ti. (**c**) EDS elemental maps of Sr. (**d**) EDS elemental maps of Mn.

**Figure S2. XRD testing of samples prepared at different deposition temperatures.**

**Figure S3. XPS test spectrum of the device.** (**a**) XPS full-spectrum peak map of HZO ferroelectric memristors prepared at 850 °C. (**b**) XPS core spectra of Hf elements. (**c**) XPS core spectra of Zr elements.

**Figure S4. The EDS characterization of surface.** (**a**) Surface morphology of the HZO film. (**b-g**) Mapping results for the elements Hf, Zr, La, Sr, Mn and O. (**h**) Mapping results and elemental composition.

**Figure S5. Ferroelectric property testing of devices.** (**a**) Displacement current corresponding to voltage correlation. (**b**) Displacement current corresponding to frequency correlation. (**c**) Ferroelectric fatigue test. (**d**) Displacement current in relation to device fatigue.

**Figure S6. Electrical property testing of devices.** (**a**) 100 I-V test cycles of the device. (**b**) Statistics on HRS and LRS resistance of 100 I-V test. (**c**) Endurance testing of the device.

**Figure S7. The yield testing of HZO ferroelectric memristors.** I-V test results for 16 devices randomly selected from the same fabrication batch.

**Figure S8. Fitting of the conduction mechanism.** (**a**) The three stages of the I-V curve. (**b**) I-V curve fitting for HRS in the on-state. (**c**) I-V curve fitting for LRS. (**d**) I-V curve fitting for HRS in the off-state. (**e**) Schematic illustration of charge carrier distribution under different polarization directions.

**Figure S9. Conductance modulation tests of HZO ferroelectric thin film memristor.** (**a**) Conductance modulation results of the device under different pulse amplitudes. (**b**) Conductance modulation results of the device under different pulse widths. (**c**) Conductance modulation results of the device under different pulse intervals. (**d**), (**e**), and (**f**) 3D visualizations of the device conductance modulation under different pulse amplitudes, widths, and intervals.

**Figure S10. EPSC testing of HZO synaptic device.** (**a**) Current response and fitting of EPSC testing at different pulse amplitudes. (**b**) Current response and fitting of EPSC testing at different numbers of pulses. (**c**) Current response and fitting of EPSC testing at different pulse widths. (**d**) Current response and fitting of EPSC testing at different pulse intervals.

**Figure S11. The simulation of the learning mechanism.** (**a**) Statistical analysis of device current response corresponding to different numbers of pulses applied at various pulse intervals. (**b**) PPF exponential distribution and fitting. (**c**) PTP exponential distribution and fitting. (**d**) Transition from PPF to PPD behavior achieved by varying the pulse sequence interval.

**Figure S12. Refractory period behavior test.** (**a, d**) Modulation of device conductance states by pulse sequences of different amplitudes. (**b, e**) Modulation of device conductance states by pulse sequences with different intervals. (**c, f**) Modulation of device conductance states by pulse sequences with different pulse widths.

**Figure S13. Simulation of reinforcement learning behavior to achieve LTM memory effects.** (**a**) Intervals set as Δt1 = 200 ns > Δt2 = 150 ns > Δt3 = 100 ns > Δt4 = 50 ns. (**b**) Intervals set as Δt1 = Δt2 = Δt3 = Δt4 = 50 ns. (**c**) Intervals set to Δt1 = 50 ns < Δt2 = 100 ns < Δt3 = 150 ns < Δt4 = 200 ns. (**d**) Conductance changes corresponding to (**a**). (**e**) Conductance changes corresponding to (**b**). (**f**) Conductance changes corresponding to (**c**).

**Figure S14. Pulses potentiation and depression.** (**a**) Schematic diagram of pulse programming. (**b**) Synaptic potentiation and depression characteristics under different numbers of potentiating and depressing pulses. (**c**) Synaptic potentiation and depression after 5 cycles when N=5. (**d**) Synaptic potentiation and depression after 5 cycles when N=10. (**e**) Synaptic potentiation and depression after 10 cycles when N=15. (**f**), (**g**), and (**h**) Statistical distributions of the maximum conductance ΔSmax and minimum conductance Δ_Smin_.

**Figure S15. Simulation of the four basic arithmetic operations: addition, subtraction, multiplication, and division**. (a) Modulating the device’s conductive state to the value “10” using 10 positive pulses. (b) Simulation of the addition operation. (c) Simulation of the multiplication operation and the commutative law of multiplication. (d) Simulation of the subtraction operation; (e) Implementation of the division operation “10 ÷ 4 = 2…2” by combining subtraction and addition.

**Figure S16. Performance of the light-sensing unit**. (a) I-V characteristic curves of the photoresistor under different light intensities. (b) Resistance distribution of the photoresistor as a function of light intensity. (c) Pulse performance test of the adaptive light-sensing unit. (d) Variation of the terminal voltage applied to the memristor as a function of the number of adaptive pulses under different light intensities.

**Figure S17. Simulation of the light adaptation process using an array of adaptive light-sensing units**. (a) Test schematic; background ambient brightness is 1 W. (b) Initial current response of the unit array under bright conditions. (c) Image perception process under illumination intensities of 1.5 W and 3.0 W.

**Figure S18. Performance of the temperature-sensing unit**. (a) I-V characteristic curve of a thermistor. (b) Resistance distribution of a thermistor at different temperatures. (c) Pulse performance test of the adaptive temperature-sensing unit. (d) Variation in the terminal voltage applied to the memristor at different ambient temperatures.

**Figure S19. Simulation of human sweating based on an adaptive temperature-sensing unit**. (**a**) Schematic diagram of human sweating, illustrating the four factors that influence sweating: ambient temperature, exercise intensity, exercise duration, and ambient humidity. (**b**) Extended pulse width simulates the effect of exercise duration on sweating. (**c**) Varying the time interval of adaptive pulses simulates the effect of ambient humidity on the human body.

**Figure S20. The three aspects of pain perception: threshold, sensitization, and desensitization**. (**a**) Threshold behavior of nociceptors. (**b**) Simulation of threshold behavior after increasing the pulse width to 2 μs. (**c**) Sensitization behavior of nociceptors, with a conditioning pulse amplitude of 0.5 V and a pulse width of 2 μs. (**d**) Desensitization process of nociceptors. (**e**) Statistics on the sensitization index corresponding to (c). (**f**) Statistics on the desensitization index corresponding to (**d**).

**Figure S21. Performance of the pressure-sensing unit**. (a) I-V characteristic curves of the thin-film pressure sensor under different applied pressures. (b) Resistance distribution of the pressure sensor at different pressures. (c) Pulse performance test; (b) Changes in the terminal voltage across the memristor following a change in pressure.

**Figure S22.** Pain sensitization index (I_v_/I_0_ × 100%, I_0_ = 0.241 μA).

**Figure S23. Multimodal adaptive sensing unit circuit based on Hf₀.₃Zr₀.₇O₂ ferroelectric thin-film memristor**. (a) and (b) are Photographs of the breadboard and circuit connections.

**Figure S24.** (a) The ratio of the system current response to the background current at a temperature of 25 °C. (b) The ratio of the system current response to the background current at a temperature of 40 °C.

**Figure S25. Correspondence Table for Numbers 0–9 in the Current Chinese Braille System.**

**Figure S26.** **Pattern-Enhanced Training.** (a) Schematic diagram of the training scheme. (b) Current response of the system under different adaptive pulses at room temperature.

**Figure S27.** **The current response of the system under different adaptive pulses in a high-temperature environment.** Where the postsynaptic current I_post_ decreases, simulates the perception of Braille information by visually impaired individuals.

**Figure S28.** After entering the programming pulses corresponding to the Braille digits 0–9, observe the current response of the sensing unit.

**Figure S29.** (a) Weight distribution in the output layer. (b) Loss function during neural network training.

**Table S1.** Comparison of Braille recognition on different platforms.

| Hardware | Speed | Power consumption | Accuracy | **References** |
| --- | --- | --- | --- | --- |
| Intel Loihi | 20 ms | 30mW | 78.32% | [1] |
| Jetson Xavier NX | >5 ms | >3 W | ~97% | [1] |
| Memristor BNN | >100 μs | - | 91.25% | [2] |
| Optoelectronic memristor | >30 ms | - | 93.58% | [3] |
| PVDF polymer sensor | >5 ms | - | 90.58% | [4] |
| Optical tactile sensor | - | - | 95.23% | [5] |
| Ferroelectric memristor | 48 μs | 8.51nJ（0.18mW） | 91.65% | **This work** |

**References:**

1. Müller-Cleve S F, Fra V, Khacef L, et al. Braille letter reading: A benchmark for spatio-temporal pattern recognition on neuromorphic hardware. Frontiers in Neuroscience, 2022, 16: 951164.

2. Liu Y H, Wang J J, Wang H Z, et al. Braille recognition by E-skin system based on binary memristive neural network. Scientific Reports, 2023, 13(1): 5437.

3. Patil J, Sharmila B, Divyashree P, et al. 1WS2 Based Optoelectronic Memristor as Artificial Synapse: Integrated Neuromorphic Computing Solution for Braille Alphabet Recognition. Optical Materials, 2025: 117346.

4. Gao Z, Chang L, Ren B, et al. Enhanced braille recognition based on piezoresistive and piezoelectric dual-mode tactile sensors. Sensors and Actuators A: Physical, 2024, 366: 115000.

5. Huang L, Luo B, Zou X, et al. Research on efficient braille recognition based on the pair-U-shaped micro-nano optical fiber fingerprint-like tactile sensors. Optics & Laser Technology, 2024, 179: 111349.
